# Evaluation of the Genotoxicity of Almond Hull: Implications for Its Use as a Novel Food Ingredient

**DOI:** 10.3390/foods13091404

**Published:** 2024-05-02

**Authors:** Yuyang Yao, Juer Liu, Qiming Miao, Xinyue Zhu, Wei Hua, Na Zhang, Guangwei Huang, Xiangyang Lin, Shengquan Mi, Yanling Cheng, Roger Ruan

**Affiliations:** 1 Biochemical Engineering College, Beijing Union University, Beijing 100101, China; buuyaoyy@foxmail.com (Y.Y.); miaoqiming1997@163.com (Q.M.); zss89661@163.com (X.Z.); huawei0917@outlook.com (W.H.); 20237003@buu.edu.cn (N.Z.); msq365@hotmail.com (S.M.); 2 Center for Biorefining and Department of Bioproducts and Biosystems Engineering, University of Minnesota, St. Paul, MN 55108, USA; liux3514@umn.edu; 3 Almond Board of California, Modesto, CA 95354, USA; ghuang@almondboard.com; 4 College of Biological Science and Engineering, Fuzhou University, Fuzhou 350025, China; xylin@fzu.edu.cn

**Keywords:** genotoxicity, OECD guidelines, almond hull, food ingredients, GRAS, food safety

## Abstract

Almond hull, a substantial byproduct comprising more than half of almond fresh weight, has recently gained attention due to its functionality and sustainability benefits. Despite heightened interest, information regarding its toxicity remains limited. In order to assess its genotoxic potential, we conducted Good Laboratory Practice-compliant in vitro and in vivo studies following Organization for Economic Co-operation and Development (OECD) guidelines. No evidence of toxicity or mutagenicity was observed in a bacterial reverse mutation assay using five tester strains, evaluating almond hull at concentrations up to 5 mg/plate, with or without metabolic activation. Almond hull did not induce chromosome structural damage in a chromosome aberration assay using Chinese hamster ovary cells, nor did it cause any spermatogonial chromosomal aberration in tested male BALB/c mice. To evaluate its ability to induce DNA damage in rodents, a combined micronucleus assay was conducted in KM mice of both sexes. Almond hull was administered at doses of 1250, 2500, and 5000 mg/kg/day via gavage once daily for 2 days. No adverse effects of almond hull were observed in the micronucleus assay. Our results indicate no evidence of the genotoxic potential of almond hull administered up to the maximum concentrations of 5 g/kg, as recommended by OECD guidelines.

## 1. Introduction

Almonds (*Prunus amygdalus* Batsch), a member of the *Rosaceae* family, stand as one of the most widely cultivated and favored tree nuts globally, contributing significantly to the high rate of tree nut production on a worldwide scale [1]. With California contributing nearly 80% of the world’s almond output, the state produced a remarkable 1.2 million metric tons of almond kernels, a primary commercial product, in the 2022/2023 crop year. The cultivation of almonds, while fruitful, generates a substantial amount of byproducts, totaling 1.8 million metric tons/year of almond hulls (AHs). This amounts to 52% of the total mass, after the initial hulling stage (Almond Board of California, 2023). The substantial amount of almond byproduct is in line with the continuous growth observed in almond production over the years. Without proper management, these large volumes of almond byproducts pose environmental and economic risks [2].

AHs typically exhibit a moisture content ranging from 8% to 20%, presenting a leathery texture and stringent taste due to prolonged exposure to environmental factors and high concentrations of flavonoids [1]. They were historically under-utilized as livestock feed in California, with a prevalent practice of burning or plowing them into the fields in the 1940s [3]. The University of California’s research in 1965 demonstrated AH’s nutritive value for ruminants. A subsequent work by Aguilar et al. in 1984 expanded AH utilization as feedstuff for dairy cattle [4,5]. Incorporating AHs into the diets of dairy cows and lactating goats has shown positive effects on digestibility and milk fat percentage without adverse outcomes [6,7]. Research on laying hens revealed no significant impact on egg quality with AH inclusion, although reductions in fat and total body mass were noted [8]. Moreover, pigs fed with AHs experienced a reduction in body fat compared to the control group [9]. Administering almond hull powder to hyperlipidemic rats resulted in decreased cholesterol and triglyceride levels, along with increased plasma antioxidant capacity [10].

Beyond animal feed applications, Holtman et al. explored the bioenergy potential of soluble sugars from AHs through a laboratory-scale process involving extraction, fermentation, and anaerobic digestion [11]. Numerous studies have investigated the abundance of phytochemicals in AHs, emphasizing their potential value [1,12,13,14,15]. Almond hulls (AHs) demonstrate stability in sugar, flavonoid, and lignan content throughout senescence, with their dry condition and moisture content being influential factors in these characteristics. Apart from soluble compounds, AHs also contain insoluble fiber, including cellulose, hemicellulose, pectin, tannin-like complex polyphenols, and ash. Three triterpenoids, betulinic, ursolic, and oleanolic acids, represent approximately 1% of an AH’s mass. Flavonol glycosides, phenolic acids, and specific compounds such as catechin, protocatechuic acid, vanillic acid, p-hydroxybenzoic acid, and naringenin glucoside are also present [16,17,18]. AHs phenolic compound compositions include phenolic acids, flavonoids, anthocyanins, lignins, and related compounds. AH extracts exhibit a total phenolic content surpassing that of kernels across different almond genotypes. These are comparable to the phenolic content in almond skins, which are considered to be the most thoroughly characterized almond byproduct and a significant source of phenolic compounds [19]. Therefore, almond hulls have significant potential applications due to their substantial nutritional value. For instance, phenolic-rich extracts from almond hulls have been proven to exhibit a protective effect, mitigating oxidative stress in Caco-2 cells [20]. Additionally, optimal conditions of extraction can yield maximum almond hull pectin (26.32% *w*/*w*) and phenolic compound levels [21]. Almond hulls boast a high total dietary fiber content, ranging from approximately 46.3% to 57.9%, enhancing their functionality in terms of water-holding capacity and emulsifying capacity [22].

However, research on the safety profile of almond hulls is limited, despite the reassuring safety that has been demonstrated by powders from the assessed varieties (*Butte*, *Monterey*, and *Nonpareil*) in our previous research [23]. While the 14-day acute oral toxicity study confirms the non-toxic or unclassified nature of these powders, there has been no prior evaluation of genotoxicity for almond hulls. In this context, the goal of the present study is to conduct a comprehensive genotoxic evaluation. A bacterial reverse mutation assay using five *Salmonella* typhimurium strains was employed as an initial screen for genotoxic activity. This will address, in particular, any mutation-inducing activity, which follows the OECD-471 guidelines [24]. Additionally, the in vitro chromosome aberration test was conducted in the CHO cell line, adhering to OECD 473 guidelines [25]. To identify if almond hulls cause structural chromosome aberrations, a mammalian in vivo micronucleus test (OECD 474) was conducted to detect potential damage to chromosomes or the mitotic apparatus of erythroblasts [26]. Furthermore, a spermatogonial chromosomal aberration test (OECD 483) was carried out using spermatocytes of BALB/c male mice [27]. All studies were conducted following OECD guidelines under Good Laboratory Practice (GLP)-compliant conditions, and the research adhered to the most recent guidelines for applying Generally Recognized as Safe (GRAS) status.

## 2. Materials and Methods

### 2.1. Chemicals and Reagents

All genotoxicity assays were conducted according to GLPs. All the chemicals and reagents utilized in this study were of analytical grade and sourced from various suppliers. Specifically, sodium azide was purchased from Tianjin Fuchen Chemical Reagent Factory (Tianjin, China); 1,8-Dihydroxyanthraquinone and 2-Aminofluorene were purchased from Sigma Aldrich (Shanghai, China); diclofenac was purchased from Chemservice (Beijing, China); cyclophosphamide monohydrate was purchased from Tishxi Ai Chemical Industrial Development Co., Ltd. (Shanghai, China); potassium dihydrogen phosphate, sodium dihydrogen phosphate, acetic acid, and trisodium citrate were purchased from Shanghai Macklin Biochemical Co., Ltd. (Shanghai, China); carboxymethyl cellulose was purchased from Shanghai Aladdin Biochemical Technology Co., Ltd. (Shanghai, China); Giemsa stain solution and the CCK-8 Assay Kit were acquired from Beijing Dingguo Changsheng Biotechnology Co., Ltd. (Beijing, China); methanol was purchased from Tianjin Damao Chemical Reagent Factory (Tianjin, China); fetal bovine serum was purchased from Zhejiang Tianhang Biological Technology Co., Ltd. (Huzhou, China); colchicine was purchased from Beijing Beten Kang Biological Pharmaceutical Technology Co., Ltd. (Beijing, China); cycloheximide was purchased from Shanghai Haoyuan Biotechnology Co., Ltd. (Shanghai, China); rat liver S9 activation system was purchased from Beijing Huizhiheyuan Biotechnology Co., Ltd. (Beijing, China); and DMEM high-glucose basal medium, PM150, PM210, 1× PBS buffer, 0.25% trypsin solution, and fetal bovine serum FBS were purchased from Procell Life Science and Technology Co., Ltd. (Wuhan, China). The *Salmonella* typhimurium histidine-deficient strains TA97, TA98 (4666D), TA100, TA102, and TA1535 were procured from Moltox (Boone, NC, USA). Distilled water was used for all animal experiments, while ultrapure water was used otherwise. 

### 2.2. Preparation of Test Item

#### 2.2.1. Almond Hull Powder

The Monterey (MT) almond hull sample, harvested in 2021 from Harris Woolf Almonds in Coalinga, CA, USA, was generously provided by the Almond Board of California. Upon receipt, the raw hull samples were stored at −20 °C in a freezer until further processing. Sample preparation involved the removal of undesirable materials such as shells, sticks, nuts, and stalks. The raw almond hulls were then rinsed twice with cold tap water. Following the rinsing process, the hulls were dried in a conventional oven at 60 °C for 48 h. After drying, the hulls underwent two rounds of grinding, using a Wiley mill (3379-K05, Thomas Scientific, Chadds Fort, NJ, USA) with a 1 mm screen to achieve fine particles. The resulting fine particles were sifted through a 100 mesh (149 μm) laboratory sieve post-milling. The processed sample AH was sealed and refrigerated until it was ready for use.

#### 2.2.2. Almond Hull Extract

The procedure for preparing almond hull extracts (AHEs) from AHs was adapted from prior research [20]. Extractions were carried out using a high-pressure microfluidizer (HPM, M110-Y, Microfluidics Corp. Westwood, MA, USA). A portion of almond powder (100 g) was combined with a mixed-solvent (1.5 L ethanol, 1.5 L water, and 3 mL acetic acid) and allowed to stand for 30 min. Subsequently, the samples underwent processing using the HPM at 152 MPa for three cycles, and the resulting material was collected in glass vials. Then, the solvent-material slurry was transferred to a 2 L conical flask in an ultrasonic cleaning bath (3510R-MTH, Branson, Danbury, CT, USA) operating at a specified ultrasonic power (330 W) for 2 h. The mixture was then subjected to centrifugation (1759× *g*, 5 min, 4 °C), and the supernatant was collected. To finalize the extraction process, ethanol and acetic acid were removed via evaporation. The extracts were subsequently freeze-dried and stored in the dark at −20 °C until analysis.

### 2.3. Animals

This animal study was approved by the Ethics Committee of the Health Food Function Testing Center of the Arts and Science College, Beijing Union University, China (Approval Code: No. 20220901). This study was conducted in accordance with the U.S. FDA Good Laboratory Practice (GLP) regulations, issued under Part 58. Title 21. Code of Federal Regulations. Specific Pathogen-Free (SPF)-grade Kunming (KM) mice and SPF BALB/c mice (Sibeifu Experiment Animal Technology Co., Ltd., Beijing, China) (Certificate: SCXK 2019-0008), weighing 25~35 g and being at an age of 7~12 weeks at the time of treatment and weighing 24~30 g and being 8~10 weeks of age at the time of treatment, respectively, were used. Animals were housed in appropriately sized polycarbonate cages with stainless steel covers, with regular ventilation in a controlled environment (12 h daily light and dark cycle, room temperature of 22 ± 2 °C, and relative humidity of 40~60%). Cage padding was replaced every three days. A pelleted diet (Beijing HFK Bioscience, Beijing, China) and sterilized drinking water were provided ad libitum. At the commencement of the experiment, animals of the same sex were weight-matched (±20%). The animals were fed for five days and subjected to observation.

### 2.4. Bacterial Reverse Mutation Assay

The methodology designed for this study was in accordance with OECD Guideline 471 [24]. Salmonella was chosen for its well-understood genetics, sensitivity to mutagens, metabolic resemblance to humans, and ethical suitability [28]. Mutagenicity assays of AHs, with and without metabolic activation, were conducted using five strains of *Salmonella typhimurium* bacteria (TA98, TA100, TA1535, TA1537, and TA102). All tested strains were purchased from Molecular Toxicology, Inc. (USA), and they were checked for the maintenance of genetic markers. Induced rat liver post-mitochondrial (S9) fraction was prepared from Wistar rats induced with Aroclor 1254 via a sterility test; the protein content (Lowry method) was <40 mg/mL. The 10 mL of S9 mixture was prepared as follows: 6.0 mL of phosphate buffer, 0.4 mL of KCL solution, 1.0 mL of glucose-6-phosphate sodium salt solution, 1.6 mL of NADP^+^, and 1.0 mL of S9 fraction. The test group design is listed in Table 1. The AH dose groups (5000 μg/plate, 1580 μg/plate, 500 μg/plate, 158 μg/plate, and 50 μg/plate) were prepared by dissolving AHs in distilled water, sterilized under 0.103 MPa for 20 min, and 0.1 mL of each dosage, 0.1 mL of bacteria solution, and 0.5 mL of 10% S9 mixture (if with metabolic activation) were added to each dose group. The blank control group was added with 0.1 mL of bacteria solution. The negative control group was added with 0.1 mL of bacteria solution and 0.1 mL of distilled sterile water. The solvent control group was added with 0.1 mL of bacteria solution and 0.1 mL of sterile dimethyl sulfoxide (DMSO). The positive control group was added with 0.1 mL of bacteria solution and 0.1 mL of positive control. Dexon (50 μg/plate) was the positive control for TA97, TA98, and TA102 without S9 mixture; sodium azide (SA) (1.5 μg/plate) was the positive control for TA100 and TA1535. Also, 2-Aminofluorene (2-AF) (10 μg/plate) was the positive control for TA97, TA98, and TA100 with S9 mixture; danthron (DAN) (50 μg/plate) was the positive control for TA102 cyclophosphamide (CP) (200 μg/plate) was the positive control for TA1535. The above test, or control article, was added to 2 mL of molten selective top agar (maintained at 45 ± 2 °C). Then, the mixture was vortexed and overlaid onto the surface of a 25 mL minimal bottom agar. After the overlay became solidified, the plates were inverted. Triplicate plates were performed for each group. The number of revertant colonies was recorded upon incubation at 37 °C for 48 h.

In response to negative test results, supplementary confirmation experiments were conducted, employing a fivefold dosage interval with concentrations of 8, 40, 200, 1000, and 5000 μg/plate. The test substance, with a highest concentration working solution of 50 mg/mL (equivalent to 5000 μg/plate), was prepared using sterile water, which was acquired under a pressure of 0.103 MPa for 20 min. Subsequent dilution with sterile water yielded doses of 1000 μg/plate, 200 μg/plate, 40 μg/plate, and 8 μg/plate, and the experimental procedures were consistent with those outlined in the previous test. 

### 2.5. Chromosome Aberration Assay

The procedures used in this study comply with OECD Guideline 473 [25]. The Chinese hamster ovary cells (CHO) were obtained from Wuhan Servicebio Technology CO., LTD (Wuhan, China) and were checked routinely to ensure the stability of the modal chromosome numbers and mycoplasma-free status. The results of a pre-toxicity test recommended a dosage of AHE up to 5000 μg/mL for this study. Test concentrations (AHEs) were formulated, ranging from 1250 to 2500 and 5000 μg/mL, and dissolved in the base culture medium. CP (0.06 μg/mL) was used as a positive control with metabolic activation (+S9); mitomycin C (MMC) at 0.5 μg/mL was used as a positive control without metabolic activation (−S9). Exposure durations were 4 and 24 h, in the absence of metabolic activation, and 4 h in the presence of metabolic activation provided by the 10% S9 mixture described above. Subsequently, approximate 1 × 10^6^ cells/mL were cultured in standard tissue culture 60 mm dishes at 37 °C in a humidified atmosphere of 5% CO_2_. Test articles were administered in cultured cells after 24 h. After 4 h of exposure to the test substance and cells, the culture medium was discarded for both the +S9/4 h and −S9/4 h groups. After gentle removal and washing with a small amount of balanced salt solution, 5 mL of culture medium containing fetal bovine serum was added. The cells were then further cultured for another 20 h; test articles remained on the continuous treatment cultures for the entire 24 h period. Colchicine was added at a concentration of 0.2 μg/mL for the final 2 h of incubation, prior to the cell harvest. At the end of the culture period, spent medium from each dish was collected; the cells were washed with PBS and harvested with 0.25% trypsin, and the cells were then incubated in a 0.075 mol/L KCl solution for 4 min at 37 °C. Then, they were fixed three times with ice-cold methanol/glacial acetic acid (3:1, *v*/*v*). The fixed cell suspension was dropped on a cold glass slide and air-dried. Slides were stained with 5% Giemsa solution and encoded. Structural and numerical chromosome damages of at least 300 metaphases per substance were observed at 1000× magnification. The percentage of cells with structural chromosomal aberrations were evaluated, and chromatid- and chromosome-type aberrations including breaks and exchanges were listed separately; gaps were recorded and reported but excluded from the total aberration frequency. 

### 2.6. In Vivo Micronucleus Test 

The principles of the OECD guideline 474 [26] were followed to perform the in vivo mammalian erythrocyte micronucleus (MN) test. According to the guideline, a single dose of up to 2000 mg/kg may be sufficient for the maximum tolerated dosage (MTD). Based on the previous study [23], the principles of both OECD 474 and OECD 423 were integrated to design the dosage for this experiment, where the maximum dosage was determined at 5000 mg/kg. Therefore, dosages of 5000, 2500, and 1250 mg/kg per body weight (BW) were used. A total of 108 KM mice (54 female and 54 male) were randomly divided into five groups: three treatment groups (*n* = 24), a positive control group (n = 12), and a negative control group (*n* = 24). Each group consisted of an equal number of male and female animals. The test articles’ AH was dissolved in 0.2% carboxymethyl cellulose (CMC) solution prior to administration. The test article and negative control (0.2% CMC) dosing formulations were orally administered daily for 2 days with a 24-h interval using stomach tubes and plastic syringes. The maximum gavage volume was 20 mL/kg∙BW. The positive control, CP, was dissolved in distilled water (80 mg/kg∙BW) for intraperitoneal injection. Following the OECD-474 sample collection method, animals were treated with the test substance once, and bone marrow samples were collected twice, starting no earlier than 24 h after the administration of the test substance but not exceeding 48 h. The sampling procedure for the negative control group was the same as that for the test substance group, while the positive control group was sampled once, specifically at 48 h. During sampling, mice underwent cervical dislocation, and bilateral femurs were quickly removed for fixation and staining using Giemsa, according to established methods [29]. Three slides were prepared for the bone marrow cells of each mouse for microscopic inspection. At least 2000 polychromatic erythrocytes (PCE) per animal were analyzed for the micronucleated ratio using a fluorescent microscope. At least 300 erythrocytes were analyzed for the relative proportions of polychromatic erythrocytes (PCE) and normochromatic erythrocytes (NCE). Thus, cytotoxicity was evaluated via the PCE/NCE ratio and PCE/PCE + NCE ratio.

### 2.7. Mammalian Spermatogonial Chromosomal Aberration Test

The mouse primary spermatocyte chromosome aberration test adapted from OECD guideline 483 was conducted to investigate the genotoxicity of AHs [27]. Similar with the OECD 474, the limit dose was 2000 mg/kg/BW/day. Thus, the MTD was determined at 5000 mg/kg/BW. A total of 54 BALB/c male mice were assigned randomly to five groups: three treatment groups (*n* = 12), a positive control group (*n* = 6), and a negative control group (*n* = 12). Dosages of 5000, 2500, and 1250 mg/kg were used. AHs were dissolved in 0.2% CMC with different concentrations, 0.2% CMC was set as solvent control, and they were administered once daily for 5 consecutive days via oral gavage at 10 mL/kg∙BW. CP was dissolved in distilled water as a positive control, and mice in the positive group were intraperitoneally injected once with 80 mg/kg∙BW of CP. Animals were treated with the test substance once, and spermatocyte samples were collected twice, at 24 and 48 h, after administering the test substance. The sampling plan for the negative control group was the same as that for the test substance group, while the positive control group was sampled once, specifically at 24 h. Three to four hours before euthanasia, a 5 mg/kg∙BW colchicine injection was administered intraperitoneally. During sampling, mice were euthanized via cervical dislocation, and both testes were removed. Subsequently, the convoluted seminiferous tubules were isolated. They then underwent hypotonic treatment, fixation, centrifugation, slide preparation, and staining; three slides were created for germ cell analysis from each mouse. Three hundred primary spermatocytes per mouse were analyzed for chromosomal aberration under the microscope, and the aberration ratio was calculated.

### 2.8. Statistical Analysis

All data were reported as the mean ± standard deviation. Results were statistically analyzed by using one-way analysis of variance (ANOVA), followed by the Tukey–Kramer test through SPSS software version 24.0 (SPSS, Inc., Chicago, IL, USA). Differences were considered significant at * *p* < 0.05, ** *p* < 0.01, and *** *p* < 0.001, respectively.

## 3. Results

### 3.1. Bacterial Reverse Mutation Test

The bacterial reverse mutation test was employed to assess the mutagenic potential of *S. typhimurium* through treatment with *S. typhimurium* histidine–auxotrophic strains. Table 2 and Table S1 present the results, indicating that, for each dose of AH within the concentration range of 50–5000 μg per plate and 8–5000 μg per plate, there was no statistically significant increase in the number of revertant colonies. This included all tested *S. typhimurium* strains (TA97, TA98, TA100, TA102, and TA1535), regardless of the presence or absence of S9 metabolic activation. These observations were made in comparison to the control, sterile water control, or sterile DMSO control groups (Table 2 and Table S1). In contrast, the positive control group exhibited pronounced mutagenic activity, with a number of revertant colonies (Dexon, SA, 2-AF, DAN, or CP) displaying a tenfold increase compared to the other groups. Consequently, it can be inferred that AH demonstrates no discernible mutagenic activity, even at the highest dose of 5000 μg per plate. Moreover, there was no apparent dose–response relationship.

### 3.2. In Vitro Chromosomal Aberration Assay

#### 3.2.1. Cytotoxicity Evaluation and Treatment Concentration

The CCK-8 assay was used for the quantification of viable cell numbers in a proliferation/cytotoxicity test; thus, a range-finding test was performed by treating AHEs of different concentrations (0, 1250, 2500, and 5000 μg/mL) on CHO cells. The cells under the absence of S9 mixture for 48 h incubation were treated with each concentration of AHE, and the cell viability was calculated, as seen in Table S2. The maximum dosage at 5000 μg/mL of AHE with cell viability of 46.67 ± 1.37% was chosen in this study based on the fact that a 55 ± 5% cytotoxicity range, which means a 45 ± 5% cell viability, was recommended for the maximum concentration in OECD 473 [25]. Typical structural aberrations (chromatid breaks) treated with a positive control compared to a negative control are illustrated in Figure S1. The structural and numerical aberrations of all test groups were observed and calculated in the following activity.

#### 3.2.2. Chromosomal Aberration Assay

The chromosomal aberration assay was adopted to assess the potential of AHEs to cause chromosomal aberrations in Chinese hamster ovary cells. Viability and the measurements of structural and numerical chromosomal abnormalities were recorded for each triplicate culture at concentration levels from 0 to 5000 μg/mL. The results of the chromosomal aberration assay for CHO cultures exposed to AHEs are summarized in Table 3. No significant difference was found regarding the chromosomal aberration rate represented by the percentage of damaged cells. This was observed at any of the analyzed concentrations of AHE, both compared to control and with or without metabolic activation. On the contrary, the positive control chemicals, cyclophosphamide and mitomycin C, elicited significant increases (*p* < 0.01) in the percentages of metaphase cells exhibiting chromosome aberrations, in comparison to concurrent negative controls across all exposure conditions. The presented data suggest that, within the parameters evaluated in this study, AHE exhibited no toxicity and did not induce structural chromosomal damage in CHO cells.

### 3.3. In Vivo Micronucleus Test

#### 3.3.1. Mouse Bone Marrow Slides

**Figure 1:**
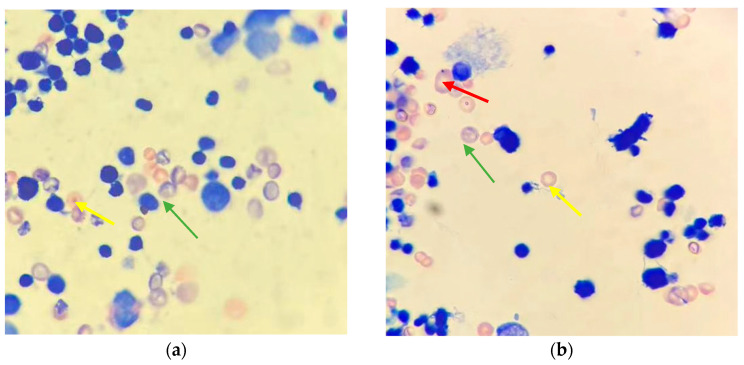

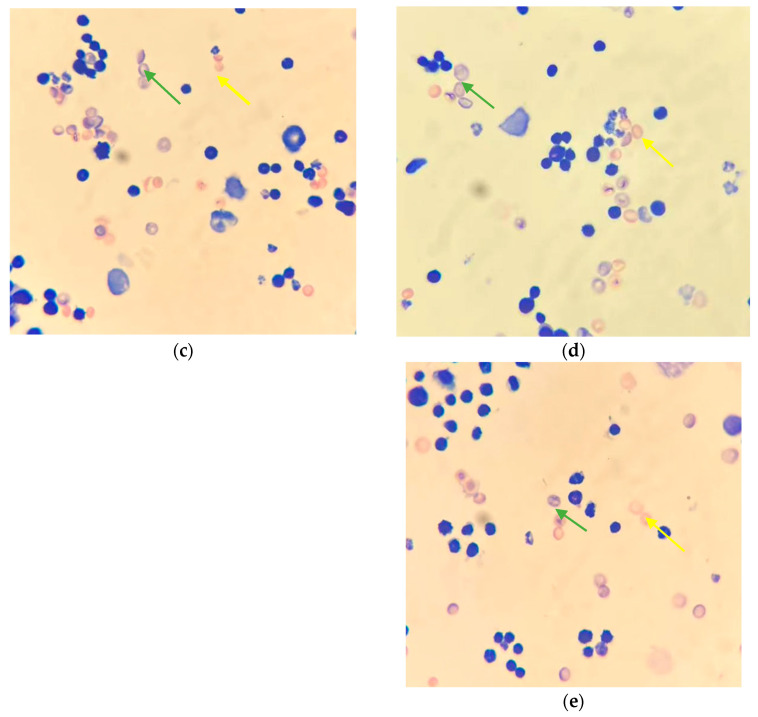
Representative photomicrographs of mouse bone marrow smear used for micronucleus analysis stained with Giemsa (magnification: ×1000): (**a**) negative control group; (**b**) positive control group; (**c**) 5000 mg/kg∙BW AH group; (**d**) 2500 mg/kg∙BW AH group; (**e**) 1250 mg/kg∙BW AH group. The red arrows indicate micronuclei in polychromatic erythrocytes (PCE), which are characterized by a circular shape, smooth and regular edges, consistent staining with the cytoplasm, and a darker color. The green arrows indicate PCE, appearing bluish-gray, while the yellow arrows indicate normochromatic erythrocytes (NCE), displaying a pink color.

No reported mortalities or signs of toxicity were observed following the administration of the test item in the conducted study. The mammalian erythrocyte micronucleus test involves analyzing animal bone marrow to detect chromosomal damage in mature red blood cells or damage to the mitotic apparatus, leading to the formation of micronuclei containing lagging chromosomal fragments or entire chromosomes. This occurrence is typically a result of exposure to chromosomal breakage agents. Additionally, in the presence of spindle poisons, the main nucleus may fail to form, giving way to a group of smaller nuclei, which are slightly larger than the typical micronuclei. In general, a micronucleus incidence rate of less than 0.5% in the negative control group suggests no damage to mammalian bone marrow cells. As shown in Figure 1, the red arrows indicate micronuclei in polychromatic erythrocytes (PCE), which are characterized by a circular shape, smooth and regular edges, consistent staining with the cytoplasm, and a darker color. The green arrows indicate PCE, appearing bluish-gray, while the yellow arrows indicate normochromatic erythrocytes (NCE), displaying a pink color. According to the results of this study, it is suggested that the almond hulls from Monterey (Mt) did not cause damage to mouse bone marrow cells.

#### 3.3.2. Mouse Bone Marrow Micronucleus Assay

No significant difference was observed in the PCE/NCE ratio between the groups treated with AHs at all tested concentrations or the negative control groups in either sex, as indicated in Table 4. Among all the groups at the 24-h and 48-h sampling points, compared to the negative control group, male mice in the high-dose (5000 mg/kg∙BW) group showed statistical differences in their micronucleated PCE (MNPCE) ratios (*p* < 0.05). The positive control groups in mice of both sexes, at both 24 h and 48 h sampling points, displayed statistically significant differences in their PCE micronucleus rates (*p* < 0.001), PCE/NCE (*p* < 0.01), and PCE/(PCE + NCE) (*p* < 0.01).

### 3.4. Mammalian Spermatogonial Chromosomal Aberration Test

#### 3.4.1. Body Weight

No mortality or unexpected clinical signs were observed in any of the groups throughout this study. The individual bodyweights of the animals at the start and end of the tests were measured and recorded. From Table S3, no significant difference is found in the body weights of the mice before oral gavage and before dissection. 

#### 3.4.2. Chromosomal Aberrations in Mouse Spermatogonia

**Figure 2:**
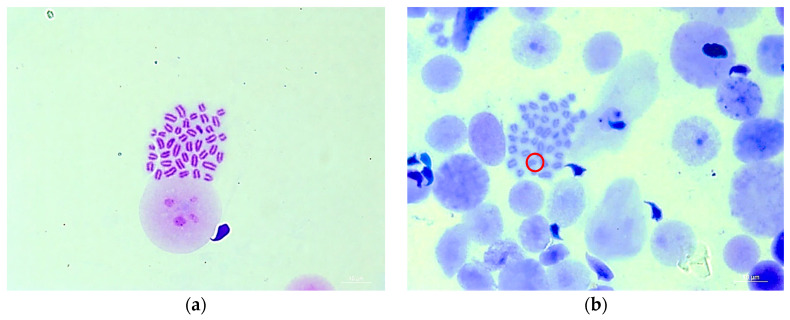
Representative photomicrographs of chromosomal aberrations in mouse spermatogonia under fluorescent microscope (magnification: ×1000): (**a**) normal metaphase of mouse spermatogonia; (**b**) mouse spermatogonia with chromatid break (as shown in red circle) treated with positive control (80 mg/kg∙BW CP).

As seen in Figure 2, it is evident that the chromosomes of mouse spermatogonia are well dispersed, making them easy to observe and identify. (a) represents normal spermatogonia chromosomes with no abnormalities, while (b) shows abnormal spermatogonia chromosomes characterized by structural chromosomal breaks (highlighted by the red circle).

#### 3.4.3. Chromosome Aberration Test in Mouse Primary Spermatocytes 

As illustrated in Table 5, the aberration ratio of spermatocytes significantly increased in the positive control group compared to the solvent control group (*p* < 0.001). However, no statistically significant differences were observed in the chromosomal aberration ratios of spermatocytes between the three different dose groups (1250 mg/kg∙BW, 2500 mg/kg∙BW, and 5000 mg/kg∙BW) and the solvent control group. In conclusion, AH, at all tested doses, demonstrated no mutagenic activity on mouse primary spermatocytes, indicating its non-genotoxic nature.

## 4. Discussion

The assessment of genotoxic potential is crucial for the registration of new food ingredients intended for human consumption. In order to support the regulatory acceptance of almond hulls in food products, the mutagenic and chromosomal aberration-inducing properties of almond hulls were comprehensively evaluated through a battery of standard in vitro and in vivo genetic toxicology assays, in accordance with OECD guidelines 471, 473, 474, and 483.

The dose design for these assays was derived from the previous acute toxicity test, with a maximum dose of 5 g/kg, and the OECD guidelines for the mammalian erythrocyte micronucleus test and mammalian spermatogonial chromosomal aberration test, which specified a limit dose of 2 g/kg. The test doses were, thus, designed at 5, 2.5, and 1.25 g/kg, equivalent to a 70 kg adult, using a conversion factor of 12.3 for mice to humans [30].

In the bacterial reverse mutation assay (Ames test), almond hulls (AH) did not elicit a positive response in any of the five test strains, and no evidence of toxicity was observed at any dose, with or without metabolic activation. Tested at concentrations up to 5 mg/plate, the consistently negative results suggest that AH is not mutagenic in the Ames test under the given conditions. No guanine–cytosine (G-C) site base-pair substitutions or frameshift mutations were detected, and there were no observed adenine–thymine (A-T) site base-pair substitutions or cross-linking mutations.

In the in vitro chromosome aberration assay using CHO cells, applied to identify potential mammalian mutagens and carcinogens, CHO cells treated with almond hull extract (AHE) were first tested for cell viability. The results showed no structural or numerical chromosomal abnormalities in CHO cells treated with AHE concentrations of 5, 2.5, and 1.25 g/kg with or without metabolic activation.

The mouse bone marrow micronucleus assay, employed to detect chemically induced chromosomal damage, revealed no systemic toxicity or clinical symptoms with almond hull treatment. Exposure to the highest dose at 5 g/kg BW of AH resulted in a substantial increase in the number of micronucleated cells compared to the vehicle control; however, it was significantly lower than that of the positive control. The bone marrow cell cytotoxicity was measured through the determination of the PCE/NCE ratio [31]. The absence of a significant difference in the PCE/NCE and PCE/PCE/+NCE ratios suggests that the genotoxic insult primarily affected the chromosomal integrity of erythrocytes without severely compromising overall erythrocyte production in the bone marrow. The interpretation of these findings is essential within the context of the specific genotoxic agent being tested and the observed dose–response relationship. In contrast, the positive control group exhibited a significantly higher development of micronuclei than the negative control group, leading to the inhibition of bone marrow cell proliferation. The data indicate that almond hulls did not induce micronuclei at the tested dose up to 5 g/kg∙BW/day.

Additionally, in the spermatogonial chromosomal aberration test, no significant difference in the number of chromosome aberrations or changes in chromosome structure or the rate of aberrant cells in the spermatogonia of mice were observed in three different dose groups of almond hulls (5000, 2500, and 1250 mg/kg BW) compared with the negative control group. There was no dose–response relationship or statistical significance among the dose groups. The chromosomal aberration cell rate difference between the cyclophosphamide positive control group and the negative control group was more significant (*p* < 0.001). According to OECD 483, within the concentration level, the results of this study show that almond hulls do not have an aberrant effect on spermatogonia chromosomes in mice, and the results are negative.

## 5. Conclusions

In summary, almond hull, evaluated up to concentrations of 5 g/kg, exceeding the maximum levels recommended by OECD guidelines, consistently yielded negative results under the conditions tested across the entire battery of genotoxicity assays conducted. Therefore, this study concludes that the application of almond hull did not induce genotoxicity in either in vitro or in vivo experiments, suggesting that almond hull may be considered safe in terms of genotoxicity. However, based on these outcomes, further research is warranted to comprehensively understand the sub-chronic toxicity profiles of almond hull through repeated dose studies in animal models. These additional investigations are crucial for completing the toxicological screenings required before almond hull can be utilized in products manufactured for human consumption.

## Figures and Tables

**Table 1:** Test Group Designs for Bacteria Reverse Mutation Tests.

Groups			Dose (μg/plate)	Bacteria Strains
Dose	±S9	0.5 mg/mL	50	TA97, TA9, TA100, TA102, TA1535
		1.58 mg/mL	158	
		5.0 mg/mL	500	
		15.8 mg/mL	1580	
		50.0 mg/mL	5000	
Positive Control	−S9	Dexon	50	TA97, TA98, TA102
		SA	1.5	TA100, TA1535
	+S9	2-AF	10	TA97, TA98, TA100
		Dan	50	TA102
		CP	200	TA1535
Negative Control	±S9	Sterile distilled water	/	TA97, TA98, TA100, TA102, TA1535
		Sterile DMSO	/	
		Blank	/	

**Table 2:** Revertant colonies in the absence or presence of S9 mix in the first bacterial reverse mutation assay results.

	Dose (μg/plate)	TA97	TA98	TA100	TA102	TA1535
Negative Control						
Sterile distilled water −S9	0	111 ± 6	35 ± 4	130 ± 12	282 ± 24	16 ± 5
Sterile distilled water +S9	0	114 ± 6	35 ± 3	136 ± 26	278 ± 30	20 ± 4
Sterile distilled DMSO −S9	0	120 ± 15	41 ± 3	161 ± 14	267 ± 23	17 ± 2
Sterile distilled DMSO +S9	0	124 ± 13	40 ± 7	159 ± 24	275 ± 19	18 ± 4
Blank −S9	0	120 ± 14	34 ± 3	137 ± 21	265 ± 17	15 ± 7
Blank +S9	0	115 ± 15	35 ± 4	141 ± 32	276 ± 12	17 ± 6
AH −S9	50	128 ± 14	37 ± 5	142 ± 21	291 ± 9	18 ± 5
	158	121 ± 11	40 ± 3	156 ± 28	271 ± 15	19 ± 4
	500	108 ± 4	40 ± 4	143 ± 23	286 ± 29	19 ± 6
	1580	125 ± 13	35 ± 6	155 ± 20	259 ± 29	16 ± 4
	5000	122 ± 7	39 ± 5	142 ± 17	284 ± 13	19 ± 6
AH +S9	50	121 ± 8	36 ± 5	144 ± 14	266 ± 22	20 ± 5
	158	112 ± 11	35 ± 4	153 ±18	275 ± 25	12 ± 2
	500	120 ± 13	39 ± 6	139 ± 23	290 ± 22	15 ± 3
	1580	114 ± 14	36 ± 3	160 ± 33	278 ± 19	21 ± 3
	5000	118 ± 16	34 ± 2	149 ± 25	286 ± 32	19 ± 3
Positive Control						
Dexon	50.0	1866 ± 198 ***	768 ± 70 ***		816 ± 96 ***	
SA	1.5			993 ± 116 ***		556 ± 37 ***
2-AF	10.0	1554 ± 75 ***	2099 ± 175 ***	960 ± 105 ***		
DAN	50.0				800 ± 86 ***	
CP	200.0					489 ± 10 ***

Data are expressed as means ± standard deviations (*n* = 3). *** Significantly different from the sterile water control group, *p* < 0.001.

**Table 3:** Summary of chromosomal aberrations by AHEs with or without S9 activation results.

Dose (μg/mL)		Chromosomal Aberration% (Without Gap)	Chromosomal Aberration (%)			Chromatid Aberration (%)		
			Break	Exchange	Gap	Break	Exchange	Gap
+S9/4 h	0	0.11 ± 0.16	0.00 ± 0.00	0.00 ± 0.00	0.00 ± 0.00	0.11 ± 0.16	0.00 ± 0.00	0.22 ± 0.16
	1250	0.11 ± 0.16	0.00 ± 0.00	0.11 ± 0.16	0.00 ± 0.00	0.00 ± 0.00	0.00 ± 0.00	0.00 ± 0.00
	2500	0.11 ± 0.16	0.00 ± 0.00	0.00 ± 0.00	0.00 ± 0.00	0.11 ± 0.16	0.00 ± 0.00	0.11 ± 0.16
	5000	0.11 ± 0.16	0.00 ± 0.00	0.00 ± 0.00	0.00 ± 0.00	0.11 ± 0.16	0.00 ± 0.00	0.56 ± 0.57
	CP	11.11 ± 0.42 **	3.33 ± 1.19	1.56 ± 0.57	2.11 ± 1.03	4.56 ± 1.26	1.67 ± 0.54	5.33 ± 1.19
−S9/4 h	1250	0.00 ± 0.00	0.00 ± 0.00	0.00 ± 0.00	0.11 ± 0.16	0.00 ± 0.00	0.00 ± 0.00	0.22 ± 0.31
	2500	0.00 ± 0.00	0.00 ± 0.00	0.00 ± 0.00	0.22 ± 0.16	0.00 ± 0.00	0.00 ± 0.00	0.11 ± 0.16
	5000	0.00 ± 0.00	0.00 ± 0.00	0.00 ± 0.00	0.00 ± 0.00	0.00 ± 0.00	0.00 ± 0.00	0.00 ± 0.00
−S9/24 h	0	0.00 ± 0.00	0.00 ± 0.00	0.00 ± 0.00	0.00 ± 0.00	0.00 ± 0.00	0.00 ± 0.00	0.00 ± 0.00
	1250	0.11 ± 0.16	0.11 ± 0.16	0.00 ± 0.00	0.00 ± 0.00	0.00 ± 0.00	0.00 ± 0.00	0.56 ± 0.16
	2500	0.22 ± 0.31	0.00 ± 0.00	0.00 ± 0.00	0.11 ± 0.16	0.22 ± 0.31	0.00 ± 0.00	0.22 ± 0.31
	5000	0.11 ± 0.16	0.00 ± 0.00	0.00 ± 0.00	0.11 ± 0.16	0.11 ± 0.16	0.00 ± 0.00	0.22 ± 0.16
	MMC	14.11 ± 0.31 **	4.33 ± 1.19	1.56 ± 0.42	5.56 ± 1.85	6.11 ± 1.81	2.11 ± 0.16	2.78 ± 0.83

Data are expressed as means ± standard deviations (*n* = 3). Structural (break in and exchange of chromatid or chromosome), and numerical aberrations were counted in 300 CHO cells. ** *p* < 0.01 compared to the negative control (0 μg/mL).

**Table 4:** Bone marrow micronucleus test in KM mice with AH results.

Groups	Dose	Sampling Point	PCE Number	MNPCE (%)	PCE/NCE	PCE/(PCE + NCE)
	(mg/kg∙BW)	(h)			(Mean ± SD)	
Male						
Solvent control	0	24	2000 × 6	0.01 ± 0.02	1.24 ± 0.19	0.55 ± 0.04
		48	2000 × 6	0.03 ± 0.04	1.23 ± 0.36	0.54 ± 0.05
Positive control (CP)	80	48	2000 × 6	6.48 ± 0.87 ^###^	0.38 ± 0.04 ^##^	0.27 ± 0.02 ^##^
AH	1250	24	2000 × 6	0.08 ± 0.11	1.19 ± 0.26	0.54 ± 0.06
		48	2000 × 6	0.02 ± 0.04	1.05 ± 0.19	0.51 ± 0.04
	2500	24	2000 × 6	0.03 ± 0.06	1.20 ± 0.24	0.54 ± 0.05
		48	2000 × 6	0.09 ± 0.13	1.22 ± 0.13	0.54 ± 0.03
	5000	24	2000 × 6	0.24 ± 0.11 *	0.93 ± 0.26	0.46 ± 0.07
		48	2000 × 6	0.26 ± 0.13 ^#^	1.11 ± 0.17	0.52 ± 0.04
Female						
Solvent control	0	24	2000 × 6	0.19 ± 0.40	1.21 ± 0.16	0.54 ± 0.03
		48	2000 × 6	0.04 ± 0.05	1.27 ± 0.19	0.56 ± 0.04
Positive control (CP)	80	48	2000 × 6	6.57 ± 0.72 ^###^	0.39 ± 0.04 ^##^	0.28 ± 0.02 ^##^
AH	1250	24	2000 × 6	0	1.07 ± 0.17	0.51 ± 0.04
		48	2000 × 6	0.02 ± 0.04	1.20 ± 0.17	0.54 ± 0.05
	2500	24	2000 × 6	0.03 ± 0.04	1.18 ± 0.24	0.54 ± 0.05
		48	2000 × 6	0.03 ± 0.04	1.13 ± 0.21	0.53 ± 0.05
	5000	24	2000 × 6	0.10 ± 0.07	1.10 ± 0.29	0.52 ± 0.06
		48	2000 × 6	0.05 ± 0.05	1.09 ± 0.17	0.52 ± 0.04

Data are expressed as means ± standard deviations (*n* = 6). * indicates statistical significance (*p* < 0.05) compared to the control at 24 h; ^#^ indicates statistical significance (*p* < 0.05) compared to the control at 48 h; ^##^ indicates statistical significance (*p* < 0.01) compared to the control at 48 h; ^###^ indicates statistical significance (*p* < 0.001) compared to the control at 48 h.

**Table 5:** Spermatogonial chromosomal aberration test in BALB/c mice treated with AH results.

Groups	Dose (mg/kg∙BW)	Sampling Point	Animals	Midterm Cells	Abnormalities Count	Chromatid		Chromosome		Fragment	Micronucleus	Chromosome Aberration (Including Gaps)	Chromosome Aberration (Excluding Gaps)
						Break	Gap	Break	Gap				
Negative control	0	24 h	6	1829	19	12	2	4	0	1	0	1.04 ± 0.25	0.93 ± 0.24
		48 h	6	1821	17	7	4	6	0	0	0	0.93 ± 0.32	0.71 ± 0.44
Positive control	80	24 h	6	1824	1036	512	149	275	48	56	17	56.80 ± 3.40 ***	46.01 ± 3.33 ***
AH	1250	24 h	6	1822	24	13	2	5	3	2	0	1.37 ± 0.37	1.09 ± 0.26
		48 h	6	1822	19	9	4	5	0	1	0	1.04 ± 0.39	0.82 ± 0.34
	2500	24 h	6	1811	14	4	4	4	0	2	0	0.77 ± 0.34	0.55 ± 0.27
		48 h	6	1835	15	7	2	5	1	0	0	0.82 ± 0.17	0.65 ± 0.20
	5000	24 h	6	1888	22	4	3	8	0	5	0	1.17 ± 0.43	1.01 ± 0.37
		48 h	6	1832	23	11	1	4	0	7	0	1.26 ± 0.37	1.21 ± 0.26

Data are expressed as mean ± standard deviation (*n* = 6). *** indicates statistical significance (*p* < 0.001) compared to the control.

## Data Availability

The original contributions presented in the study are included in the article/Supplementary Materials, further inquiries can be directed to the corresponding authors.

## Supplementary Materials

The following supporting information can be downloaded via this link: https://www.mdpi.com/article/10.3390/foods13091404/s1, Figure S1: Representative photomicrographs of chromosomal aberrations in CHO cells under fluorescent microscope; Table S1: Revertant colonies in the absence or presence of S9 mix in the second bacterial reverse mutation assay results; Table S2: Cytotoxicity of AHE against CHO cells; Table S3: Body weight of BALB/c mice before gavage and dissection for the spermatogonial chromosomal assay.
