# Supplementary material for: Evaluation of the Genotoxicity of Almond Hull: Implications for Its Use as a Novel Food Ingredient
Source: Foods. 2024 May 2;13(9):1404. doi: 10.3390/foods13091404 (PMC11083516; doi:10.3390/foods13091404)
Supplement: Supplementary file 1 [file foods-13-01404-s001.zip › foods-2979719-supplementary.pdf]

Table S1. Revertant colonies in the absence or presence of S9 mix in the second bacterial reverse mutation assay results.

	Dose (μg/plate)	TA97	TA98	TA100	TA102	TA1535
Negative Control						
Sterile distilled water −S9	0	122 ± 13	40 ± 4	152 ± 23	265 ± 10	17 ± 6
Sterile distilled water +S9	0	125 ± 5	39 ± 4	138 ± 15	257 ± 14	19 ± 7
Sterile distilled DMSO −S9	0	110 ± 9	37 ± 4	154 ± 18	256 ± 20	17 ± 4
Sterile distilled DMSO +S9	0	118 ± 2	36 ± 6	150 ± 10	276 ± 14	19 ± 5
Blank −S9	0	109 ± 13	39 ± 4	163 ± 11	284 ± 29	21 ± 2
Blank +S9	0	122 ± 10	40 ± 4	149 ± 11	273 ± 19	20 ± 5
AH −S9	8	118 ± 8	39 ± 4	162 ± 11	292 ± 16	18 ± 7
	40	116 ± 11	31 ± 2	143 ± 29	268 ± 16	17 ± 5
	200	114 ± 15	39 ± 3	159 ± 17	279 ± 16	12 ± 3
	1000	115 ± 10	35 ± 4	156 ± 16	256 ± 12	22 ± 4
	5000	116 ± 14	40 ± 5	140 ± 30	301 ± 8	14 ± 1
AH +S9	8	121 ± 16	35 ± 5	145 ± 29	270 ± 14	17 ± 6
	40	128 ± 1	34 ± 3	157 ± 27	294 ± 13	12 ± 1
	200	125 ± 15	34 ± 1	152 ± 13	278 ± 24	15 ± 3
	1000	118 ± 4	37 ± 6	148 ± 24	265 ± 14	16 ± 7
	5000	109 ± 9	36 ± 7	153 ± 20	275 ± 30	17 ± 5
Positive Control						
Dexon	50.0	1833 ± 81 ***	833 ± 110 ***		753 ± 46 ***	
SA	1.5			922 ± 89 ***		515 ± 60 ***
2-AF	10.0	1478 ± 70 ***	2023 ± 99 ***	967 ± 100 ***		
DAN	50.0				850 ± 46 ***	
CP	200.0					436 ± 51 ***

Data are expressed as means ± standard deviations (n = 3). *** Significantly different from the sterile water control group, $p < 0.001$.

Table S2. Cytotoxicity of AHE against CHO cells.

Dose (μg/mL)	Cell Viability (%)
0	100.00 ± 0.00
1250	88.73 ± 1.90
2500	66.73 ± 3.57
5000	46.67 ± 1.37

Data a expressed as means ± standard deviations (n = 3).

Table S3. Body weight of BALB/c mice before gavage and dissection for the spermatogonial chromosomal assay.

Groups	Dose (mg/kg·BW)	Samling Point	Body Weight (g)	
			Before Gavage	Before Dissection
Negative control	0	24	26.22 ± 0.67	26.32 ± 0.67
		48	26.70 ± 0.57	26.93 ± 0.59
Positive control (CP)	80	24	26.32 ± 1.19	26.40 ± 1.18
AH	1250	24	26.02 ± 1.03	26.08 ± 1.09
		48	26.07 ± 1.23	26.25 ± 1.30
	2500	24	26.13 ± 1.02	26.22 ± 1.09
		48	26.03 ± 1.05	26.20 ± 1.08
	5000	24	26.13 ± 0.56	26.17 ± 0.57
		48	26.30 ± 0.47	26.41 ± 0.45

Data are expressed as means ± standard deviations (n = 6).

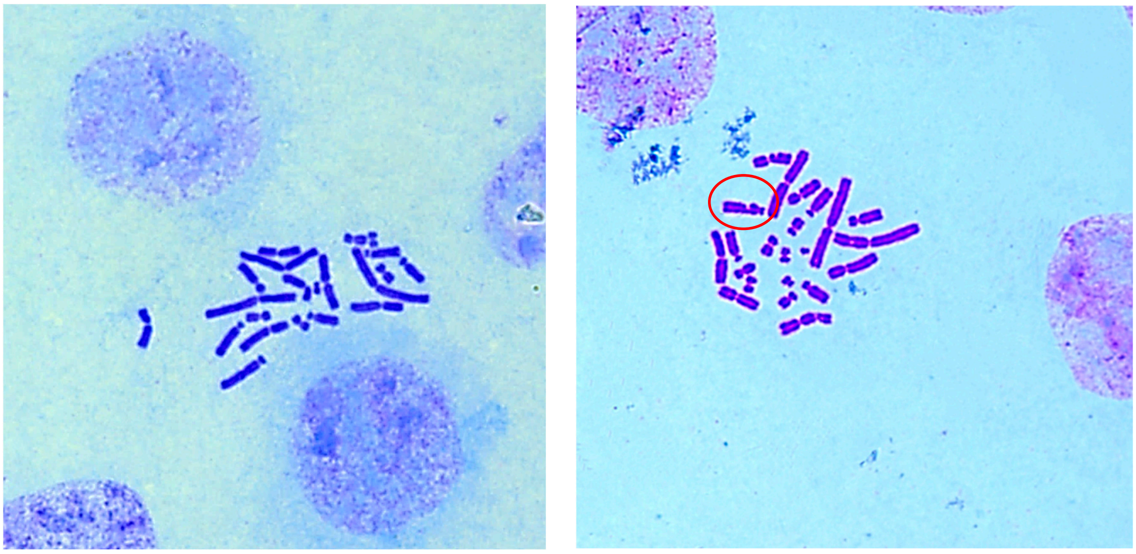

(**a**) (**b**)

Figure S1. Representative photomicrographs of chromosomal aberrations in CHO cells under fluorescent microscope (magnification: ×1000): (**a**) normal metaphase of CHO cell; (**b**) CHO cell with chromatid break (as shown in red circle) treated with positive control (0.06 μg/mL CP).
